# Tribenzyl­chlorido(triphenyl­phosphine oxide-κ*O*)tin(IV)

**DOI:** 10.1107/S160053681101957X

**Published:** 2011-05-28

**Authors:** Kong Mun Lo, Seik Weng Ng

**Affiliations:** a Department of Chemistry, University of Malaya, 50603 Kuala Lumpur, Malaysia

## Abstract

In the title tribenzyl­chloridotin–triphenyl­phosphine adduct, [Sn(C_7_H_7_)_3_Cl(C_18_H_15_OP)], the Sn^IV^ atom is in a *trans*-C_3_SnClO trigonal–bipyramidal geometry and is displaced out of the C_3_Sn girdle in the direction of the axial Cl atom by 0.112 (1) in one independent mol­ecule and by 0.167 (1) Å in the other. The phenyl ring of one of the six benzyl units was refined as equally disordered over two sets of sites.

## Related literature

For the trimethyl­tin chloride–triphenyl­phosphine adduct, see: Davis *et al.* (2007 ▶). For the analogous triphenyl­tin chloride adduct, see: de Castro *et al.* (2001 ▶); Eppley *et al.* (1992 ▶); Ng & Kumar Das (1992 ▶).
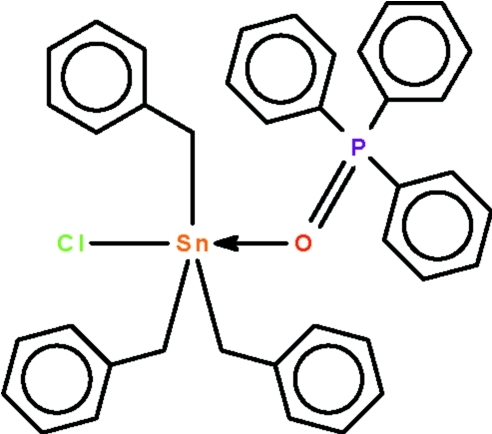


## Experimental

### 

#### Crystal data

[Sn(C_7_H_7_)_3_Cl(C_18_H_15_OP)]
*M* *_r_* = 705.81
Monoclinic, 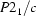

*a* = 10.0697 (1) Å
*b* = 31.8793 (4) Å
*c* = 21.3259 (3) Å
β = 100.4304 (6)°
*V* = 6732.81 (14) Å^3^
*Z* = 8
Mo *K*α radiation
μ = 0.92 mm^−1^
*T* = 100 K
0.30 × 0.25 × 0.20 mm

#### Data collection

Bruker SMART APEX diffractometer
Absorption correction: multi-scan (*SADABS*; Sheldrick, 1996 ▶) *T*_min_ = 0.771, *T*_max_ = 0.838
62453 measured reflections
15456 independent reflections
13210 reflections with *I* > 2σ(*I*)
*R*_int_ = 0.047

#### Refinement

*R*[*F* ^2^ > 2σ(*F* ^2^)] = 0.031
*wR*(*F* ^2^) = 0.072
*S* = 1.03
15456 reflections
769 parameters
37 restraints
H-atom parameters constrained
Δρ_max_ = 0.76 e Å^−3^
Δρ_min_ = −0.67 e Å^−3^

### 

Data collection: *APEX2* (Bruker, 2009 ▶); cell refinement: *SAINT* (Bruker, 2009 ▶); data reduction: *SAINT*; program(s) used to solve structure: *SHELXS97* (Sheldrick, 2008 ▶); program(s) used to refine structure: *SHELXL97* (Sheldrick, 2008 ▶); molecular graphics: *X-SEED* (Barbour, 2001 ▶); software used to prepare material for publication: *publCIF* (Westrip, 2010 ▶).

## Supplementary Material

Crystal structure: contains datablocks global, I. DOI: 10.1107/S160053681101957X/zs2112sup1.cif

Structure factors: contains datablocks I. DOI: 10.1107/S160053681101957X/zs2112Isup2.hkl

Additional supplementary materials:  crystallographic information; 3D view; checkCIF report

## Figures and Tables

**Fig. 1.**
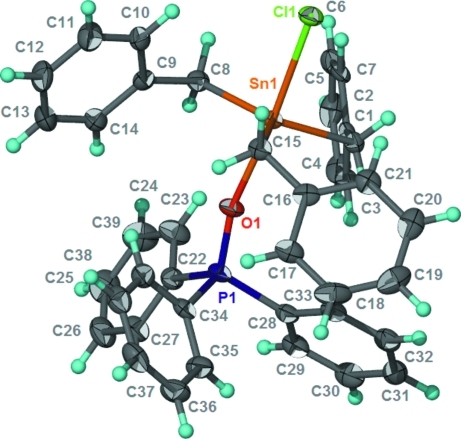
Thermal ellipsoid plot (Barbour, 2001) of one independent (C6H5CH2)3SnCl.O═P(C6H5)3 adduct at the 70% probability level. Hydrogen atoms are drawn as spheres of arbitrary radius.

**Fig. 2.**
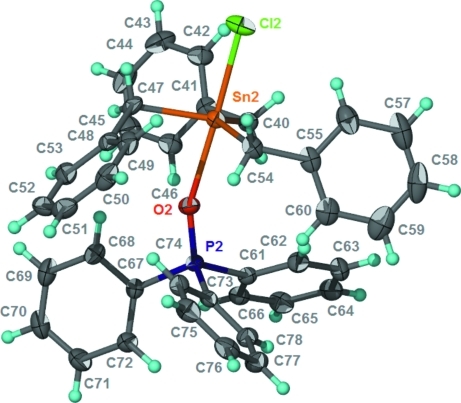
Thermal ellipsoid plot (Barbour, 2001) of the second independent (C6H5CH2)3SnCl.O═P(C6H5)3 adduct at the 70% probability level. Hydrogen atoms are drawn as spheres of arbitrary radius.

## supplementary crystallographic information

### Comment

Triorganotin halides, being strong Lewis acids, form a large number of adducts with oxygen-donor ligands. With triphenylphosphine oxide in particular, the crystal structures of the adducts with trimethyltin chloride (Davis *et al.*, 2007) and triphenyltin chloride (de Castro *et al.*, 2001; Eppley *et al.*, 1992; Ng & Kumar Das, 1992) have been reported. The tribenzylchloridotin–triphenylphosphine adduct (Scheme I) has the Sn^IV^ atom in a *trans*-C_3_SnClO trigonal bipyramidal geometry. The Sn atom is displaced out of the C_3_Sn girdle in the direction of the axial Cl atom [by 0.112 (1) Å in one independent molecule (Fig. 1) and by 0.167 (1) Å in the other independent molecule (Fig. 2)].

### Experimental

Tribenzyltin chloride (0.42, 1 mmol) and triphenylphosphine oxide (0.28 g, 1 mmol) were heated in chloroform (20 ml) until the reactants dissolved completely. The filtered solution was set aside for the growth of colorless crystals, which separated after a day.

### Refinement

Carbon-bound H-atoms were placed in calculated positions (C—H 0.95 to 0.99 Å) and were included in the refinement in the riding model approximation, with *U*_iso_(H) set to 1.2*U*_eq_(C). The phenyl ring of one of the six benzyl units is disordered over two positions; the occupancy could not be refined, and was assumed to be a 0.5: 0.5 type of disorder. The rings were refined as rigid hexagons of 1.39 Å sides, the C_methylene_–C_phenyl_ pair of distances were restrained to within ±0.01 Å of each other, and the displacement parameters of the primed atoms were set to those of the unprimed ones. The anisotropic displacement parameters were restrained to be nearly isotropic.

### Crystal data

**Table:**

[Sn(C_7_H_7_)_3_Cl(C_18_H_15_OP)]	*F*(000) = 2880
*M**_r_* = 705.81	*D*_x_ = 1.393 Mg m^−^^3^
Monoclinic, *P*2_1_/*c*	Mo *K*α radiation, λ = 0.71073 Å
Hall symbol: -P 2ybc	Cell parameters from 9802 reflections
*a* = 10.0697 (1) Å	θ = 2.5–28.3°
*b* = 31.8793 (4) Å	µ = 0.92 mm^−^^1^
*c* = 21.3259 (3) Å	*T* = 100 K
β = 100.4304 (6)°	Block, colorless
*V* = 6732.81 (14) Å^3^	0.30 × 0.25 × 0.20 mm
*Z* = 8	

### Data collection

**Table:**

Bruker SMART APEX diffractometer	15456 independent reflections
Radiation source: fine-focus sealed tube	13210 reflections with *I* > 2σ(*I*)
graphite	*R*_int_ = 0.047
ω scans	θ_max_ = 27.5°, θ_min_ = 1.9°
Absorption correction: multi-scan (*SADABS*; Sheldrick, 1996)	*h* = −13→12
*T*_min_ = 0.771, *T*_max_ = 0.838	*k* = −41→41
62453 measured reflections	*l* = −27→27

### Refinement

**Table:**

Refinement on *F*^2^	Primary atom site location: structure-invariant direct methods
Least-squares matrix: full	Secondary atom site location: difference Fourier map
*R*[*F*^2^ > 2σ(*F*^2^)] = 0.031	Hydrogen site location: inferred from neighbouring sites
*wR*(*F*^2^) = 0.072	H-atom parameters constrained
*S* = 1.03	*w* = 1/[σ^2^(*F*_o_^2^) + (0.0298*P*)^2^ + 4.2265*P*] where *P* = (*F*_o_^2^ + 2*F*_c_^2^)/3
15456 reflections	(Δ/σ)_max_ = 0.001
769 parameters	Δρ_max_ = 0.76 e Å^−^^3^
37 restraints	Δρ_min_ = −0.67 e Å^−^^3^

### Fractional atomic coordinates and isotropic or equivalent isotropic displacement parameters (Å^2^)

**Table:**

	*x*	*y*	*z*	*U*_iso_*/*U*_eq_	Occ. (<1)
Sn1	0.745692 (13)	0.778572 (4)	0.606789 (7)	0.01401 (4)	
Sn2	0.223618 (13)	0.512678 (4)	0.776274 (7)	0.01688 (4)	
Cl1	0.91606 (5)	0.764087 (17)	0.70807 (3)	0.02120 (11)	
Cl2	0.06400 (5)	0.53153 (2)	0.84882 (3)	0.02915 (13)	
P1	0.49337 (5)	0.801792 (18)	0.45899 (3)	0.01764 (12)	
P2	0.43088 (5)	0.490489 (17)	0.64836 (3)	0.01656 (11)	
O1	0.58536 (15)	0.78523 (5)	0.51702 (7)	0.0214 (3)	
O2	0.36436 (15)	0.48998 (5)	0.70558 (8)	0.0213 (3)	
C1	0.6501 (2)	0.82902 (6)	0.64850 (11)	0.0176 (4)	
H1A	0.7135	0.8406	0.6854	0.021*	0.50
H1B	0.6264	0.8517	0.6168	0.021*	0.50
H1C	0.7078	0.8377	0.6890	0.021*	0.50
H1D	0.6390	0.8534	0.6193	0.021*	0.50
C2	0.5231 (7)	0.8136 (5)	0.6703 (6)	0.0146 (9)	0.50
C3	0.3967 (9)	0.8271 (4)	0.6397 (6)	0.0218 (9)	0.50
H3	0.3895	0.8466	0.6056	0.026*	0.50
C4	0.2809 (6)	0.8122 (3)	0.6592 (4)	0.0297 (14)	0.50
H4	0.1945	0.8215	0.6383	0.036*	0.50
C5	0.2915 (5)	0.7837 (3)	0.7092 (3)	0.0302 (17)	0.50
H5	0.2123	0.7736	0.7224	0.036*	0.50
C6	0.4179 (6)	0.7702 (3)	0.7397 (3)	0.0252 (13)	0.50
H6	0.4251	0.7507	0.7739	0.030*	0.50
C7	0.5337 (4)	0.7851 (4)	0.7203 (5)	0.0179 (12)	0.50
H7	0.6201	0.7758	0.7412	0.022*	0.50
C2'	0.5136 (6)	0.8155 (5)	0.6611 (6)	0.0146 (9)	0.50
C3'	0.3951 (9)	0.8342 (4)	0.6301 (6)	0.0218 (9)	0.50
H3'	0.3986	0.8553	0.5989	0.026*	0.50
C4'	0.2716 (7)	0.8221 (3)	0.6447 (5)	0.0297 (14)	0.50
H4'	0.1906	0.8349	0.6234	0.036*	0.50
C5'	0.2666 (5)	0.7913 (3)	0.6903 (3)	0.0302 (17)	0.50
H5'	0.1822	0.7830	0.7003	0.036*	0.50
C6'	0.3851 (6)	0.7726 (2)	0.7213 (3)	0.0252 (13)	0.50
H6'	0.3817	0.7515	0.7525	0.030*	0.50
C7'	0.5086 (5)	0.7847 (4)	0.7067 (5)	0.0179 (12)	0.50
H7'	0.5896	0.7719	0.7280	0.022*	0.50
C8	0.6655 (2)	0.71581 (6)	0.60576 (11)	0.0192 (4)	
H8A	0.6813	0.7043	0.6496	0.023*	
H8B	0.5670	0.7163	0.5897	0.023*	
C9	0.7327 (2)	0.68875 (6)	0.56402 (11)	0.0192 (4)	
C10	0.8666 (2)	0.67583 (7)	0.58421 (12)	0.0220 (5)	
H10	0.9137	0.6846	0.6248	0.026*	
C11	0.9316 (2)	0.65072 (7)	0.54676 (13)	0.0289 (5)	
H11	1.0222	0.6422	0.5618	0.035*	
C12	0.8653 (3)	0.63771 (8)	0.48707 (13)	0.0316 (6)	
H12	0.9097	0.6203	0.4612	0.038*	
C13	0.7334 (3)	0.65053 (8)	0.46588 (12)	0.0323 (6)	
H13	0.6874	0.6420	0.4250	0.039*	
C14	0.6678 (2)	0.67567 (7)	0.50365 (12)	0.0251 (5)	
H14	0.5774	0.6842	0.4883	0.030*	
C15	0.9002 (2)	0.79212 (7)	0.55210 (11)	0.0193 (4)	
H15A	0.9886	0.7827	0.5762	0.023*	
H15B	0.8815	0.7762	0.5116	0.023*	
C16	0.90819 (19)	0.83807 (7)	0.53749 (11)	0.0195 (5)	
C17	0.8965 (2)	0.85230 (8)	0.47475 (11)	0.0239 (5)	
H17	0.8843	0.8327	0.4407	0.029*	
C18	0.9026 (2)	0.89505 (8)	0.46176 (13)	0.0323 (6)	
H18	0.8943	0.9044	0.4189	0.039*	
C19	0.9207 (2)	0.92386 (8)	0.51074 (14)	0.0325 (6)	
H19	0.9241	0.9530	0.5016	0.039*	
C20	0.9340 (2)	0.91035 (8)	0.57303 (14)	0.0287 (6)	
H20	0.9475	0.9301	0.6069	0.034*	
C21	0.9278 (2)	0.86774 (7)	0.58619 (12)	0.0224 (5)	
H21	0.9370	0.8587	0.6292	0.027*	
C22	0.3438 (2)	0.76998 (7)	0.44126 (11)	0.0206 (5)	
C23	0.3007 (2)	0.74912 (8)	0.49142 (12)	0.0292 (5)	
H23	0.3522	0.7509	0.5333	0.035*	
C24	0.1823 (3)	0.72574 (9)	0.47991 (14)	0.0367 (6)	
H24	0.1528	0.7114	0.5140	0.044*	
C25	0.1072 (3)	0.72329 (8)	0.41898 (15)	0.0365 (7)	
H25	0.0266	0.7072	0.4112	0.044*	
C26	0.1490 (2)	0.74414 (8)	0.36953 (13)	0.0338 (6)	
H26	0.0965	0.7425	0.3278	0.041*	
C27	0.2671 (2)	0.76754 (8)	0.37998 (12)	0.0261 (5)	
H27	0.2955	0.7818	0.3456	0.031*	
C28	0.4403 (2)	0.85444 (7)	0.46948 (10)	0.0196 (4)	
C29	0.3045 (2)	0.86599 (8)	0.46075 (11)	0.0240 (5)	
H29	0.2358	0.8458	0.4477	0.029*	
C30	0.2704 (2)	0.90737 (8)	0.47138 (12)	0.0291 (5)	
H30	0.1781	0.9155	0.4651	0.035*	
C31	0.3696 (3)	0.93652 (8)	0.49099 (12)	0.0281 (5)	
H31	0.3456	0.9647	0.4983	0.034*	
C32	0.5051 (2)	0.92508 (7)	0.50027 (11)	0.0256 (5)	
H32	0.5732	0.9454	0.5142	0.031*	
C33	0.5408 (2)	0.88433 (7)	0.48930 (11)	0.0217 (5)	
H33	0.6333	0.8766	0.4952	0.026*	
C34	0.5789 (2)	0.80212 (7)	0.39232 (10)	0.0191 (4)	
C35	0.5664 (2)	0.83505 (8)	0.34867 (11)	0.0256 (5)	
H35	0.5041	0.8571	0.3513	0.031*	
C36	0.6445 (3)	0.83575 (8)	0.30150 (12)	0.0300 (5)	
H36	0.6358	0.8583	0.2719	0.036*	
C37	0.7348 (2)	0.80389 (8)	0.29743 (12)	0.0286 (5)	
H37	0.7895	0.8048	0.2655	0.034*	
C38	0.7462 (2)	0.77050 (8)	0.33961 (12)	0.0271 (5)	
H38	0.8075	0.7483	0.3361	0.033*	
C39	0.6690 (2)	0.76942 (7)	0.38665 (12)	0.0233 (5)	
H39	0.6768	0.7464	0.4154	0.028*	
C40	0.0634 (2)	0.49702 (7)	0.69772 (11)	0.0196 (4)	
H40A	−0.0178	0.5140	0.7000	0.024*	
H40B	0.0923	0.5031	0.6567	0.024*	
C41	0.0315 (2)	0.45124 (7)	0.70179 (10)	0.0184 (4)	
C42	−0.0702 (2)	0.43763 (7)	0.73324 (12)	0.0255 (5)	
H42	−0.1229	0.4577	0.7508	0.031*	
C43	−0.0958 (3)	0.39515 (8)	0.73924 (13)	0.0330 (6)	
H43	−0.1661	0.3865	0.7606	0.040*	
C44	−0.0199 (3)	0.36541 (8)	0.71439 (12)	0.0326 (6)	
H44	−0.0370	0.3364	0.7190	0.039*	
C45	0.0811 (3)	0.37840 (8)	0.68280 (13)	0.0311 (6)	
H45	0.1338	0.3582	0.6655	0.037*	
C46	0.1058 (2)	0.42074 (7)	0.67619 (12)	0.0244 (5)	
H46	0.1747	0.4292	0.6538	0.029*	
C47	0.3230 (2)	0.46353 (7)	0.83715 (11)	0.0224 (5)	
H47A	0.2881	0.4635	0.8777	0.027*	
H47B	0.3008	0.4361	0.8162	0.027*	
C48	0.4739 (2)	0.46884 (7)	0.85147 (11)	0.0199 (5)	
C49	0.5328 (2)	0.50544 (7)	0.87912 (11)	0.0246 (5)	
H49	0.4766	0.5273	0.8897	0.030*	
C50	0.6715 (2)	0.51072 (8)	0.89150 (12)	0.0301 (6)	
H50	0.7093	0.5363	0.9092	0.036*	
C51	0.7549 (2)	0.47891 (9)	0.87814 (13)	0.0329 (6)	
H51	0.8502	0.4824	0.8867	0.040*	
C52	0.6986 (2)	0.44201 (9)	0.85232 (12)	0.0314 (6)	
H52	0.7555	0.4197	0.8439	0.038*	
C53	0.5594 (2)	0.43704 (8)	0.83842 (11)	0.0253 (5)	
H53	0.5221	0.4116	0.8198	0.030*	
C54	0.3345 (2)	0.57118 (7)	0.78922 (12)	0.0223 (5)	
H54A	0.3424	0.5795	0.8345	0.027*	
H54B	0.4271	0.5655	0.7818	0.027*	
C55	0.2812 (2)	0.60816 (7)	0.74926 (12)	0.0240 (5)	
C56	0.1711 (2)	0.63098 (8)	0.76282 (14)	0.0316 (6)	
H56	0.1258	0.6218	0.7957	0.038*	
C57	0.1275 (3)	0.66700 (8)	0.72840 (16)	0.0412 (8)	
H57	0.0532	0.6824	0.7382	0.049*	
C58	0.1913 (3)	0.68039 (8)	0.68034 (16)	0.0430 (8)	
H58	0.1607	0.7050	0.6569	0.052*	
C59	0.3001 (3)	0.65814 (8)	0.66595 (14)	0.0399 (7)	
H59	0.3442	0.6674	0.6326	0.048*	
C60	0.3445 (2)	0.62220 (7)	0.70057 (13)	0.0291 (6)	
H60	0.4193	0.6071	0.6907	0.035*	
C61	0.3140 (2)	0.50362 (7)	0.57643 (11)	0.0188 (4)	
C62	0.2527 (2)	0.54308 (7)	0.57332 (11)	0.0230 (5)	
H62	0.2761	0.5623	0.6076	0.028*	
C63	0.1583 (2)	0.55421 (7)	0.52060 (12)	0.0263 (5)	
H63	0.1171	0.5811	0.5187	0.032*	
C64	0.1233 (2)	0.52634 (8)	0.47043 (12)	0.0272 (5)	
H64	0.0592	0.5343	0.4340	0.033*	
C65	0.1815 (2)	0.48715 (8)	0.47342 (12)	0.0260 (5)	
H65	0.1564	0.4679	0.4393	0.031*	
C66	0.2774 (2)	0.47558 (7)	0.52646 (11)	0.0210 (5)	
H66	0.3175	0.4485	0.5283	0.025*	
C67	0.5046 (2)	0.44010 (7)	0.63761 (11)	0.0191 (4)	
C68	0.4809 (2)	0.40737 (7)	0.67713 (11)	0.0234 (5)	
H68	0.4242	0.4116	0.7077	0.028*	
C69	0.5402 (3)	0.36846 (7)	0.67200 (13)	0.0304 (6)	
H69	0.5253	0.3462	0.6996	0.036*	
C70	0.6202 (2)	0.36205 (8)	0.62716 (13)	0.0317 (6)	
H70	0.6597	0.3353	0.6235	0.038*	
C71	0.6435 (3)	0.39442 (8)	0.58721 (14)	0.0323 (6)	
H71	0.6985	0.3897	0.5561	0.039*	
C72	0.5872 (2)	0.43357 (7)	0.59241 (12)	0.0259 (5)	
H72	0.6045	0.4559	0.5654	0.031*	
C73	0.5639 (2)	0.52880 (6)	0.65812 (11)	0.0180 (4)	
C74	0.6488 (2)	0.53007 (7)	0.71749 (12)	0.0226 (5)	
H74	0.6355	0.5109	0.7499	0.027*	
C75	0.7525 (2)	0.55922 (8)	0.72939 (12)	0.0272 (5)	
H75	0.8110	0.5598	0.7697	0.033*	
C76	0.7707 (2)	0.58743 (7)	0.68228 (13)	0.0281 (5)	
H76	0.8411	0.6076	0.6906	0.034*	
C77	0.6869 (2)	0.58641 (7)	0.62312 (13)	0.0267 (5)	
H77	0.7001	0.6058	0.5910	0.032*	
C78	0.5839 (2)	0.55707 (7)	0.61074 (11)	0.0222 (5)	
H78	0.5269	0.5562	0.5701	0.027*	

### Atomic displacement parameters (Å^2^)

**Table:**

	*U*^11^	*U*^22^	*U*^33^	*U*^12^	*U*^13^	*U*^23^
Sn1	0.01355 (7)	0.01552 (7)	0.01280 (7)	0.00046 (5)	0.00199 (5)	0.00052 (5)
Sn2	0.01476 (7)	0.01836 (7)	0.01724 (8)	−0.00094 (5)	0.00209 (6)	−0.00184 (6)
Cl1	0.0198 (2)	0.0239 (3)	0.0176 (3)	−0.00029 (19)	−0.0027 (2)	0.0027 (2)
Cl2	0.0197 (2)	0.0408 (3)	0.0283 (3)	−0.0025 (2)	0.0080 (2)	−0.0129 (3)
P1	0.0155 (2)	0.0235 (3)	0.0129 (3)	0.0010 (2)	−0.0002 (2)	0.0011 (2)
P2	0.0175 (2)	0.0176 (3)	0.0151 (3)	0.00056 (19)	0.0045 (2)	0.0012 (2)
O1	0.0207 (7)	0.0254 (8)	0.0156 (8)	0.0029 (6)	−0.0034 (6)	0.0026 (6)
O2	0.0206 (7)	0.0256 (8)	0.0185 (8)	−0.0002 (6)	0.0059 (6)	0.0011 (7)
C1	0.0190 (9)	0.0179 (10)	0.0158 (11)	0.0001 (8)	0.0033 (8)	−0.0019 (8)
C2	0.0165 (11)	0.0155 (14)	0.011 (3)	0.0012 (11)	0.0004 (14)	−0.0034 (16)
C3	0.0232 (11)	0.022 (3)	0.018 (3)	0.0070 (15)	−0.0010 (14)	−0.0009 (16)
C4	0.0142 (13)	0.027 (4)	0.043 (4)	0.0098 (17)	−0.0069 (18)	−0.009 (3)
C5	0.023 (2)	0.026 (3)	0.044 (5)	−0.005 (2)	0.012 (3)	−0.009 (3)
C6	0.031 (3)	0.0271 (16)	0.019 (4)	−0.005 (2)	0.007 (3)	−0.001 (2)
C7	0.0181 (18)	0.0218 (12)	0.012 (4)	0.000 (2)	−0.003 (2)	−0.003 (2)
C2'	0.0165 (11)	0.0155 (14)	0.011 (3)	0.0012 (11)	0.0004 (14)	−0.0034 (16)
C3'	0.0232 (11)	0.022 (3)	0.018 (3)	0.0070 (15)	−0.0010 (14)	−0.0009 (16)
C4'	0.0142 (13)	0.027 (4)	0.043 (4)	0.0098 (17)	−0.0069 (18)	−0.009 (3)
C5'	0.023 (2)	0.026 (3)	0.044 (5)	−0.005 (2)	0.012 (3)	−0.009 (3)
C6'	0.031 (3)	0.0271 (16)	0.019 (4)	−0.005 (2)	0.007 (3)	−0.001 (2)
C7'	0.0181 (18)	0.0218 (12)	0.012 (4)	0.000 (2)	−0.003 (2)	−0.003 (2)
C8	0.0185 (10)	0.0198 (10)	0.0185 (11)	−0.0025 (8)	0.0014 (9)	0.0014 (9)
C9	0.0236 (10)	0.0145 (10)	0.0192 (11)	−0.0029 (8)	0.0032 (9)	0.0029 (9)
C10	0.0227 (10)	0.0189 (10)	0.0226 (12)	−0.0029 (8)	−0.0012 (9)	−0.0028 (9)
C11	0.0249 (11)	0.0258 (12)	0.0347 (15)	0.0008 (9)	0.0023 (10)	−0.0050 (11)
C12	0.0409 (14)	0.0253 (12)	0.0303 (14)	0.0037 (10)	0.0111 (12)	−0.0091 (11)
C13	0.0449 (14)	0.0266 (12)	0.0219 (13)	0.0008 (11)	−0.0034 (11)	−0.0063 (10)
C14	0.0273 (11)	0.0226 (11)	0.0224 (13)	0.0013 (9)	−0.0032 (10)	−0.0015 (10)
C15	0.0173 (10)	0.0226 (11)	0.0186 (11)	0.0017 (8)	0.0050 (9)	0.0002 (9)
C16	0.0098 (9)	0.0263 (11)	0.0229 (12)	0.0001 (8)	0.0038 (8)	0.0040 (9)
C17	0.0189 (10)	0.0324 (12)	0.0205 (12)	−0.0018 (9)	0.0036 (9)	0.0041 (10)
C18	0.0226 (11)	0.0411 (15)	0.0324 (15)	−0.0038 (10)	0.0025 (10)	0.0183 (12)
C19	0.0204 (11)	0.0255 (12)	0.0512 (18)	−0.0037 (9)	0.0048 (11)	0.0118 (12)
C20	0.0169 (10)	0.0263 (12)	0.0436 (16)	−0.0023 (9)	0.0070 (10)	−0.0039 (11)
C21	0.0157 (10)	0.0277 (12)	0.0240 (12)	−0.0004 (8)	0.0041 (9)	0.0003 (10)
C22	0.0188 (10)	0.0227 (11)	0.0194 (12)	0.0014 (8)	0.0012 (9)	−0.0009 (9)
C23	0.0225 (11)	0.0401 (14)	0.0235 (13)	−0.0035 (10)	0.0005 (10)	0.0041 (11)
C24	0.0269 (12)	0.0443 (16)	0.0389 (17)	−0.0067 (11)	0.0060 (12)	0.0093 (13)
C25	0.0222 (12)	0.0379 (14)	0.0474 (18)	−0.0073 (10)	0.0006 (12)	−0.0036 (13)
C26	0.0280 (12)	0.0382 (14)	0.0311 (15)	−0.0018 (10)	−0.0054 (11)	−0.0097 (12)
C27	0.0235 (11)	0.0330 (13)	0.0209 (12)	0.0005 (9)	0.0017 (9)	−0.0031 (10)
C28	0.0212 (10)	0.0241 (11)	0.0130 (11)	0.0027 (8)	0.0019 (8)	0.0016 (9)
C29	0.0208 (10)	0.0321 (12)	0.0189 (12)	0.0015 (9)	0.0030 (9)	0.0006 (10)
C30	0.0255 (11)	0.0398 (14)	0.0227 (13)	0.0116 (10)	0.0062 (10)	0.0002 (11)
C31	0.0405 (13)	0.0268 (12)	0.0188 (12)	0.0085 (10)	0.0101 (10)	−0.0006 (10)
C32	0.0322 (12)	0.0289 (12)	0.0162 (12)	−0.0019 (10)	0.0055 (10)	0.0020 (10)
C33	0.0212 (10)	0.0278 (12)	0.0160 (11)	0.0009 (9)	0.0027 (9)	0.0029 (9)
C34	0.0173 (9)	0.0265 (11)	0.0124 (11)	−0.0009 (8)	0.0000 (8)	−0.0014 (9)
C35	0.0284 (11)	0.0289 (12)	0.0199 (12)	0.0065 (9)	0.0051 (10)	0.0038 (10)
C36	0.0381 (13)	0.0347 (14)	0.0182 (13)	0.0013 (11)	0.0081 (11)	0.0059 (10)
C37	0.0253 (11)	0.0421 (14)	0.0192 (12)	−0.0029 (10)	0.0062 (10)	−0.0070 (11)
C38	0.0229 (11)	0.0335 (13)	0.0248 (13)	0.0042 (9)	0.0040 (10)	−0.0068 (11)
C39	0.0232 (11)	0.0239 (11)	0.0219 (12)	0.0010 (9)	0.0019 (9)	−0.0019 (9)
C40	0.0172 (9)	0.0224 (11)	0.0186 (11)	−0.0008 (8)	0.0017 (8)	0.0004 (9)
C41	0.0178 (9)	0.0221 (11)	0.0132 (11)	−0.0010 (8)	−0.0030 (8)	−0.0025 (9)
C42	0.0261 (11)	0.0278 (12)	0.0240 (13)	−0.0045 (9)	0.0078 (10)	−0.0065 (10)
C43	0.0440 (14)	0.0332 (14)	0.0243 (14)	−0.0148 (11)	0.0123 (12)	−0.0031 (11)
C44	0.0497 (15)	0.0202 (12)	0.0252 (14)	−0.0084 (11)	−0.0003 (12)	0.0011 (10)
C45	0.0342 (13)	0.0250 (12)	0.0324 (15)	0.0029 (10)	0.0015 (11)	−0.0068 (11)
C46	0.0232 (11)	0.0253 (12)	0.0252 (13)	−0.0021 (9)	0.0058 (9)	−0.0050 (10)
C47	0.0181 (10)	0.0317 (12)	0.0166 (11)	−0.0013 (9)	0.0009 (9)	0.0094 (10)
C48	0.0206 (10)	0.0253 (11)	0.0135 (11)	−0.0005 (8)	0.0023 (8)	0.0032 (9)
C49	0.0252 (11)	0.0270 (12)	0.0196 (12)	0.0021 (9)	−0.0013 (9)	0.0016 (10)
C50	0.0285 (12)	0.0322 (13)	0.0257 (14)	−0.0072 (10)	−0.0058 (10)	0.0070 (11)
C51	0.0190 (11)	0.0533 (17)	0.0263 (14)	−0.0002 (10)	0.0034 (10)	0.0134 (12)
C52	0.0271 (12)	0.0478 (16)	0.0210 (13)	0.0137 (11)	0.0086 (10)	0.0062 (11)
C53	0.0297 (12)	0.0280 (12)	0.0176 (12)	0.0046 (9)	0.0028 (10)	0.0008 (10)
C54	0.0189 (10)	0.0183 (10)	0.0286 (13)	−0.0019 (8)	0.0018 (9)	−0.0050 (9)
C55	0.0221 (10)	0.0201 (11)	0.0269 (13)	−0.0013 (8)	−0.0036 (9)	−0.0062 (10)
C56	0.0275 (12)	0.0280 (13)	0.0359 (15)	0.0041 (10)	−0.0033 (11)	−0.0063 (11)
C57	0.0343 (14)	0.0275 (13)	0.054 (2)	0.0082 (11)	−0.0125 (14)	−0.0084 (13)
C58	0.0448 (15)	0.0201 (12)	0.053 (2)	−0.0024 (11)	−0.0209 (15)	0.0033 (12)
C59	0.0444 (15)	0.0313 (14)	0.0382 (17)	−0.0124 (12)	−0.0075 (13)	0.0047 (12)
C60	0.0291 (12)	0.0235 (12)	0.0318 (14)	−0.0051 (9)	−0.0018 (11)	−0.0017 (10)
C61	0.0183 (9)	0.0211 (10)	0.0173 (11)	0.0004 (8)	0.0045 (8)	0.0020 (9)
C62	0.0260 (11)	0.0239 (11)	0.0191 (12)	0.0005 (9)	0.0038 (9)	−0.0015 (9)
C63	0.0252 (11)	0.0256 (12)	0.0278 (13)	0.0069 (9)	0.0039 (10)	0.0029 (10)
C64	0.0195 (11)	0.0400 (14)	0.0210 (13)	0.0036 (9)	0.0010 (9)	0.0030 (11)
C65	0.0233 (11)	0.0335 (13)	0.0206 (12)	0.0001 (9)	0.0025 (9)	−0.0063 (10)
C66	0.0203 (10)	0.0220 (11)	0.0218 (12)	0.0001 (8)	0.0069 (9)	−0.0012 (9)
C67	0.0196 (10)	0.0179 (10)	0.0191 (11)	0.0012 (8)	0.0021 (9)	0.0017 (9)
C68	0.0269 (11)	0.0250 (11)	0.0180 (12)	0.0001 (9)	0.0027 (9)	0.0032 (9)
C69	0.0359 (13)	0.0217 (12)	0.0312 (15)	0.0016 (10)	−0.0002 (11)	0.0074 (10)
C70	0.0298 (12)	0.0235 (12)	0.0398 (16)	0.0084 (9)	0.0014 (11)	0.0001 (11)
C71	0.0327 (13)	0.0272 (13)	0.0402 (16)	0.0066 (10)	0.0150 (12)	0.0000 (11)
C72	0.0278 (11)	0.0227 (11)	0.0295 (14)	0.0032 (9)	0.0115 (10)	0.0038 (10)
C73	0.0178 (9)	0.0168 (10)	0.0206 (11)	0.0024 (8)	0.0067 (9)	−0.0014 (9)
C74	0.0216 (10)	0.0230 (11)	0.0228 (12)	0.0028 (8)	0.0030 (9)	0.0012 (9)
C75	0.0199 (10)	0.0325 (13)	0.0278 (14)	0.0000 (9)	0.0004 (10)	−0.0052 (11)
C76	0.0234 (11)	0.0243 (12)	0.0381 (15)	−0.0024 (9)	0.0095 (11)	−0.0074 (11)
C77	0.0313 (12)	0.0201 (11)	0.0314 (14)	−0.0032 (9)	0.0127 (11)	0.0008 (10)
C78	0.0255 (11)	0.0215 (11)	0.0206 (12)	−0.0003 (9)	0.0067 (9)	−0.0009 (9)

### Geometric parameters (Å, °)

**Table:**

Sn1—C1	2.148 (2)	C31—C32	1.391 (3)
Sn1—C15	2.151 (2)	C31—H31	0.9500
Sn1—C8	2.156 (2)	C32—C33	1.379 (3)
Sn1—O1	2.2798 (14)	C32—H32	0.9500
Sn1—Cl1	2.5456 (5)	C33—H33	0.9500
Sn2—C40	2.163 (2)	C34—C35	1.393 (3)
Sn2—C47	2.161 (2)	C34—C39	1.402 (3)
Sn2—C54	2.165 (2)	C35—C36	1.385 (3)
Sn2—O2	2.3618 (16)	C35—H35	0.9500
Sn2—Cl2	2.4977 (6)	C36—C37	1.376 (4)
P1—O1	1.5011 (15)	C36—H36	0.9500
P1—C28	1.788 (2)	C37—C38	1.385 (4)
P1—C34	1.791 (2)	C37—H37	0.9500
P1—C22	1.798 (2)	C38—C39	1.377 (4)
P2—O2	1.4947 (17)	C38—H38	0.9500
P2—C73	1.797 (2)	C39—H39	0.9500
P2—C67	1.802 (2)	C40—C41	1.500 (3)
P2—C61	1.806 (2)	C40—H40A	0.9900
C1—C2'	1.510 (5)	C40—H40B	0.9900
C1—C2	1.520 (5)	C41—C42	1.391 (3)
C1—H1A	0.9900	C41—C46	1.397 (3)
C1—H1B	0.9900	C42—C43	1.389 (3)
C1—H1C	0.9900	C42—H42	0.9500
C1—H1D	0.9900	C43—C44	1.382 (4)
C2—C3	1.3900	C43—H43	0.9500
C2—C7	1.3900	C44—C45	1.381 (4)
C3—C4	1.3900	C44—H44	0.9500
C3—H3	0.9500	C45—C46	1.384 (3)
C4—C5	1.3900	C45—H45	0.9500
C4—H4	0.9500	C46—H46	0.9500
C5—C6	1.3900	C47—C48	1.504 (3)
C5—H5	0.9500	C47—H47A	0.9900
C6—C7	1.3900	C47—H47B	0.9900
C6—H6	0.9500	C48—C49	1.391 (3)
C7—H7	0.9500	C48—C53	1.391 (3)
C2'—C3'	1.3900	C49—C50	1.384 (3)
C2'—C7'	1.3900	C49—H49	0.9500
C3'—C4'	1.3900	C50—C51	1.379 (4)
C3'—H3'	0.9500	C50—H50	0.9500
C4'—C5'	1.3900	C51—C52	1.377 (4)
C4'—H4'	0.9500	C51—H51	0.9500
C5'—C6'	1.3900	C52—C53	1.388 (3)
C5'—H5'	0.9500	C52—H52	0.9500
C6'—C7'	1.3900	C53—H53	0.9500
C6'—H6'	0.9500	C54—C55	1.496 (3)
C7'—H7'	0.9500	C54—H54A	0.9900
C8—C9	1.487 (3)	C54—H54B	0.9900
C8—H8A	0.9900	C55—C60	1.387 (4)
C8—H8B	0.9900	C55—C56	1.400 (3)
C9—C14	1.398 (3)	C56—C57	1.390 (4)
C9—C10	1.401 (3)	C56—H56	0.9500
C10—C11	1.377 (3)	C57—C58	1.372 (5)
C10—H10	0.9500	C57—H57	0.9500
C11—C12	1.389 (3)	C58—C59	1.386 (4)
C11—H11	0.9500	C58—H58	0.9500
C12—C13	1.384 (4)	C59—C60	1.392 (4)
C12—H12	0.9500	C59—H59	0.9500
C13—C14	1.386 (4)	C60—H60	0.9500
C13—H13	0.9500	C61—C66	1.389 (3)
C14—H14	0.9500	C61—C62	1.398 (3)
C15—C16	1.503 (3)	C62—C63	1.381 (3)
C15—H15A	0.9900	C62—H62	0.9500
C15—H15B	0.9900	C63—C64	1.386 (3)
C16—C21	1.392 (3)	C63—H63	0.9500
C16—C17	1.397 (3)	C64—C65	1.376 (3)
C17—C18	1.394 (3)	C64—H64	0.9500
C17—H17	0.9500	C65—C66	1.397 (3)
C18—C19	1.378 (4)	C65—H65	0.9500
C18—H18	0.9500	C66—H66	0.9500
C19—C20	1.380 (4)	C67—C68	1.389 (3)
C19—H19	0.9500	C67—C72	1.398 (3)
C20—C21	1.391 (3)	C68—C69	1.389 (3)
C20—H20	0.9500	C68—H68	0.9500
C21—H21	0.9500	C69—C70	1.373 (4)
C22—C23	1.394 (3)	C69—H69	0.9500
C22—C27	1.394 (3)	C70—C71	1.385 (4)
C23—C24	1.390 (3)	C70—H70	0.9500
C23—H23	0.9500	C71—C72	1.384 (3)
C24—C25	1.382 (4)	C71—H71	0.9500
C24—H24	0.9500	C72—H72	0.9500
C25—C26	1.375 (4)	C73—C74	1.394 (3)
C25—H25	0.9500	C73—C78	1.395 (3)
C26—C27	1.387 (3)	C74—C75	1.387 (3)
C26—H26	0.9500	C74—H74	0.9500
C27—H27	0.9500	C75—C76	1.385 (4)
C28—C29	1.395 (3)	C75—H75	0.9500
C28—C33	1.399 (3)	C76—C77	1.385 (4)
C29—C30	1.392 (3)	C76—H76	0.9500
C29—H29	0.9500	C77—C78	1.387 (3)
C30—C31	1.373 (4)	C77—H77	0.9500
C30—H30	0.9500	C78—H78	0.9500
			
C1—Sn1—C15	119.79 (8)	C28—C29—H29	120.2
C1—Sn1—C8	120.26 (8)	C31—C30—C29	120.2 (2)
C15—Sn1—C8	119.15 (9)	C31—C30—H30	119.9
C1—Sn1—O1	88.60 (7)	C29—C30—H30	119.9
C15—Sn1—O1	89.88 (7)	C30—C31—C32	120.5 (2)
C8—Sn1—O1	82.59 (7)	C30—C31—H31	119.8
C1—Sn1—Cl1	93.91 (6)	C32—C31—H31	119.8
C15—Sn1—Cl1	93.09 (6)	C33—C32—C31	120.1 (2)
C8—Sn1—Cl1	91.94 (6)	C33—C32—H32	120.0
O1—Sn1—Cl1	174.52 (4)	C31—C32—H32	120.0
C40—Sn2—C47	119.81 (8)	C32—C33—C28	119.8 (2)
C40—Sn2—C54	126.34 (8)	C32—C33—H33	120.1
C47—Sn2—C54	112.08 (8)	C28—C33—H33	120.1
C40—Sn2—O2	83.37 (7)	C35—C34—C39	119.0 (2)
C47—Sn2—O2	84.00 (8)	C35—C34—P1	122.46 (18)
C54—Sn2—O2	89.43 (8)	C39—C34—P1	118.42 (18)
C40—Sn2—Cl2	93.55 (6)	C36—C35—C34	120.2 (2)
C47—Sn2—Cl2	94.71 (7)	C36—C35—H35	119.9
C54—Sn2—Cl2	95.08 (7)	C34—C35—H35	119.9
O2—Sn2—Cl2	175.46 (4)	C37—C36—C35	120.1 (2)
O1—P1—C28	112.39 (10)	C37—C36—H36	119.9
O1—P1—C34	110.06 (10)	C35—C36—H36	119.9
C28—P1—C34	106.95 (11)	C36—C37—C38	120.3 (2)
O1—P1—C22	110.08 (10)	C36—C37—H37	119.8
C28—P1—C22	107.31 (10)	C38—C37—H37	119.8
C34—P1—C22	109.95 (11)	C39—C38—C37	120.1 (2)
O2—P2—C73	110.35 (10)	C39—C38—H38	120.0
O2—P2—C67	110.50 (10)	C37—C38—H38	120.0
C73—P2—C67	107.75 (10)	C38—C39—C34	120.3 (2)
O2—P2—C61	112.13 (10)	C38—C39—H39	119.9
C73—P2—C61	107.37 (10)	C34—C39—H39	119.9
C67—P2—C61	108.59 (10)	C41—C40—Sn2	108.50 (13)
P1—O1—Sn1	164.27 (10)	C41—C40—H40A	110.0
P2—O2—Sn2	158.00 (9)	Sn2—C40—H40A	110.0
C2'—C1—Sn1	110.5 (7)	C41—C40—H40B	110.0
C2—C1—Sn1	110.4 (7)	Sn2—C40—H40B	110.0
C2'—C1—H1A	116.2	H40A—C40—H40B	108.4
C2—C1—H1A	109.6	C42—C41—C46	117.7 (2)
Sn1—C1—H1A	109.6	C42—C41—C40	121.0 (2)
C2'—C1—H1B	102.5	C46—C41—C40	121.3 (2)
C2—C1—H1B	109.6	C43—C42—C41	120.9 (2)
Sn1—C1—H1B	109.6	C43—C42—H42	119.5
H1A—C1—H1B	108.1	C41—C42—H42	119.5
C2'—C1—H1C	109.6	C44—C43—C42	120.6 (2)
C2—C1—H1C	102.6	C44—C43—H43	119.7
Sn1—C1—H1C	109.6	C42—C43—H43	119.7
C2'—C1—H1D	109.6	C43—C44—C45	119.2 (2)
C2—C1—H1D	116.3	C43—C44—H44	120.4
Sn1—C1—H1D	109.6	C45—C44—H44	120.4
H1C—C1—H1D	108.1	C46—C45—C44	120.3 (2)
C3—C2—C7	120.0	C46—C45—H45	119.9
C3—C2—C1	120.3 (6)	C44—C45—H45	119.9
C7—C2—C1	119.7 (7)	C45—C46—C41	121.3 (2)
C2—C3—C4	120.0	C45—C46—H46	119.3
C2—C3—H3	120.0	C41—C46—H46	119.3
C4—C3—H3	120.0	C48—C47—Sn2	112.40 (15)
C5—C4—C3	120.0	C48—C47—H47A	109.1
C5—C4—H4	120.0	Sn2—C47—H47A	109.1
C3—C4—H4	120.0	C48—C47—H47B	109.1
C4—C5—C6	120.0	Sn2—C47—H47B	109.1
C4—C5—H5	120.0	H47A—C47—H47B	107.9
C6—C5—H5	120.0	C49—C48—C53	117.7 (2)
C5—C6—C7	120.0	C49—C48—C47	121.2 (2)
C5—C6—H6	120.0	C53—C48—C47	121.1 (2)
C7—C6—H6	120.0	C50—C49—C48	121.4 (2)
C6—C7—C2	120.0	C50—C49—H49	119.3
C6—C7—H7	120.0	C48—C49—H49	119.3
C2—C7—H7	120.0	C51—C50—C49	120.2 (2)
C3'—C2'—C7'	120.0	C51—C50—H50	119.9
C3'—C2'—C1	121.5 (7)	C49—C50—H50	119.9
C7'—C2'—C1	118.4 (7)	C52—C51—C50	119.2 (2)
C2'—C3'—C4'	120.0	C52—C51—H51	120.4
C2'—C3'—H3'	120.0	C50—C51—H51	120.4
C4'—C3'—H3'	120.0	C51—C52—C53	120.7 (2)
C3'—C4'—C5'	120.0	C51—C52—H52	119.7
C3'—C4'—H4'	120.0	C53—C52—H52	119.7
C5'—C4'—H4'	120.0	C48—C53—C52	120.8 (2)
C6'—C5'—C4'	120.0	C48—C53—H53	119.6
C6'—C5'—H5'	120.0	C52—C53—H53	119.6
C4'—C5'—H5'	120.0	C55—C54—Sn2	118.86 (14)
C5'—C6'—C7'	120.0	C55—C54—H54A	107.6
C5'—C6'—H6'	120.0	Sn2—C54—H54A	107.6
C7'—C6'—H6'	120.0	C55—C54—H54B	107.6
C6'—C7'—C2'	120.0	Sn2—C54—H54B	107.6
C6'—C7'—H7'	120.0	H54A—C54—H54B	107.0
C2'—C7'—H7'	120.0	C60—C55—C56	118.4 (2)
C9—C8—Sn1	109.53 (14)	C60—C55—C54	121.0 (2)
C9—C8—H8A	109.8	C56—C55—C54	120.5 (2)
Sn1—C8—H8A	109.8	C57—C56—C55	120.4 (3)
C9—C8—H8B	109.8	C57—C56—H56	119.8
Sn1—C8—H8B	109.8	C55—C56—H56	119.8
H8A—C8—H8B	108.2	C58—C57—C56	120.4 (3)
C14—C9—C10	117.3 (2)	C58—C57—H57	119.8
C14—C9—C8	122.5 (2)	C56—C57—H57	119.8
C10—C9—C8	120.2 (2)	C57—C58—C59	120.1 (3)
C11—C10—C9	121.6 (2)	C57—C58—H58	119.9
C11—C10—H10	119.2	C59—C58—H58	119.9
C9—C10—H10	119.2	C58—C59—C60	119.7 (3)
C10—C11—C12	120.3 (2)	C58—C59—H59	120.2
C10—C11—H11	119.8	C60—C59—H59	120.2
C12—C11—H11	119.8	C55—C60—C59	121.1 (3)
C11—C12—C13	119.0 (2)	C55—C60—H60	119.5
C11—C12—H12	120.5	C59—C60—H60	119.5
C13—C12—H12	120.5	C66—C61—C62	119.3 (2)
C12—C13—C14	120.7 (2)	C66—C61—P2	123.00 (17)
C12—C13—H13	119.6	C62—C61—P2	117.60 (17)
C14—C13—H13	119.6	C63—C62—C61	120.2 (2)
C13—C14—C9	121.0 (2)	C63—C62—H62	119.9
C13—C14—H14	119.5	C61—C62—H62	119.9
C9—C14—H14	119.5	C62—C63—C64	120.3 (2)
C16—C15—Sn1	112.28 (14)	C62—C63—H63	119.8
C16—C15—H15A	109.1	C64—C63—H63	119.8
Sn1—C15—H15A	109.1	C65—C64—C63	120.0 (2)
C16—C15—H15B	109.1	C65—C64—H64	120.0
Sn1—C15—H15B	109.1	C63—C64—H64	120.0
H15A—C15—H15B	107.9	C64—C65—C66	120.2 (2)
C21—C16—C17	118.0 (2)	C64—C65—H65	119.9
C21—C16—C15	120.9 (2)	C66—C65—H65	119.9
C17—C16—C15	121.1 (2)	C61—C66—C65	120.0 (2)
C18—C17—C16	120.6 (2)	C61—C66—H66	120.0
C18—C17—H17	119.7	C65—C66—H66	120.0
C16—C17—H17	119.7	C68—C67—C72	119.7 (2)
C19—C18—C17	120.4 (2)	C68—C67—P2	118.18 (18)
C19—C18—H18	119.8	C72—C67—P2	122.07 (17)
C17—C18—H18	119.8	C69—C68—C67	120.0 (2)
C18—C19—C20	119.9 (2)	C69—C68—H68	120.0
C18—C19—H19	120.1	C67—C68—H68	120.0
C20—C19—H19	120.1	C70—C69—C68	120.2 (2)
C19—C20—C21	119.9 (2)	C70—C69—H69	119.9
C19—C20—H20	120.0	C68—C69—H69	119.9
C21—C20—H20	120.0	C69—C70—C71	120.3 (2)
C16—C21—C20	121.2 (2)	C69—C70—H70	119.9
C16—C21—H21	119.4	C71—C70—H70	119.9
C20—C21—H21	119.4	C72—C71—C70	120.3 (3)
C23—C22—C27	119.7 (2)	C72—C71—H71	119.8
C23—C22—P1	118.21 (17)	C70—C71—H71	119.8
C27—C22—P1	121.96 (19)	C71—C72—C67	119.6 (2)
C22—C23—C24	119.8 (2)	C71—C72—H72	120.2
C22—C23—H23	120.1	C67—C72—H72	120.2
C24—C23—H23	120.1	C74—C73—C78	119.6 (2)
C25—C24—C23	120.1 (3)	C74—C73—P2	116.53 (17)
C25—C24—H24	119.9	C78—C73—P2	123.84 (17)
C23—C24—H24	119.9	C75—C74—C73	120.2 (2)
C26—C25—C24	120.2 (2)	C75—C74—H74	119.9
C26—C25—H25	119.9	C73—C74—H74	119.9
C24—C25—H25	119.9	C76—C75—C74	119.8 (2)
C25—C26—C27	120.6 (2)	C76—C75—H75	120.1
C25—C26—H26	119.7	C74—C75—H75	120.1
C27—C26—H26	119.7	C75—C76—C77	120.5 (2)
C26—C27—C22	119.6 (2)	C75—C76—H76	119.8
C26—C27—H27	120.2	C77—C76—H76	119.8
C22—C27—H27	120.2	C78—C77—C76	120.0 (2)
C29—C28—C33	119.9 (2)	C78—C77—H77	120.0
C29—C28—P1	122.62 (17)	C76—C77—H77	120.0
C33—C28—P1	117.44 (16)	C77—C78—C73	119.9 (2)
C30—C29—C28	119.5 (2)	C77—C78—H78	120.0
C30—C29—H29	120.2	C73—C78—H78	120.0
			
C28—P1—O1—Sn1	−36.3 (4)	P1—C28—C33—C32	−177.74 (18)
C34—P1—O1—Sn1	82.8 (4)	O1—P1—C34—C35	−139.12 (18)
C22—P1—O1—Sn1	−155.9 (4)	C28—P1—C34—C35	−16.8 (2)
C1—Sn1—O1—P1	55.3 (4)	C22—P1—C34—C35	99.5 (2)
C15—Sn1—O1—P1	−64.5 (4)	O1—P1—C34—C39	36.24 (19)
C8—Sn1—O1—P1	176.0 (4)	C28—P1—C34—C39	158.60 (17)
C73—P2—O2—Sn2	76.2 (3)	C22—P1—C34—C39	−85.20 (18)
C67—P2—O2—Sn2	−164.7 (2)	C39—C34—C35—C36	−1.6 (3)
C61—P2—O2—Sn2	−43.4 (3)	P1—C34—C35—C36	173.77 (18)
C40—Sn2—O2—P2	67.7 (3)	C34—C35—C36—C37	0.1 (4)
C47—Sn2—O2—P2	−171.3 (3)	C35—C36—C37—C38	1.3 (4)
C54—Sn2—O2—P2	−59.0 (3)	C36—C37—C38—C39	−1.2 (4)
C15—Sn1—C1—C2'	157.5 (5)	C37—C38—C39—C34	−0.3 (3)
C8—Sn1—C1—C2'	−12.1 (5)	C35—C34—C39—C38	1.6 (3)
O1—Sn1—C1—C2'	68.4 (5)	P1—C34—C39—C38	−173.88 (17)
Cl1—Sn1—C1—C2'	−106.7 (5)	C47—Sn2—C40—C41	6.89 (19)
C15—Sn1—C1—C2	166.0 (4)	C54—Sn2—C40—C41	170.44 (14)
C8—Sn1—C1—C2	−3.6 (5)	O2—Sn2—C40—C41	86.06 (15)
O1—Sn1—C1—C2	77.0 (5)	Cl2—Sn2—C40—C41	−90.60 (15)
Cl1—Sn1—C1—C2	−98.2 (4)	Sn2—C40—C41—C42	93.2 (2)
C2'—C1—C2—C3	−20 (9)	Sn2—C40—C41—C46	−84.5 (2)
Sn1—C1—C2—C3	−112.1 (6)	C46—C41—C42—C43	0.5 (3)
C2'—C1—C2—C7	159 (10)	C40—C41—C42—C43	−177.3 (2)
Sn1—C1—C2—C7	66.7 (9)	C41—C42—C43—C44	0.4 (4)
C7—C2—C3—C4	0.0	C42—C43—C44—C45	−0.7 (4)
C1—C2—C3—C4	178.7 (13)	C43—C44—C45—C46	0.0 (4)
C2—C3—C4—C5	0.0	C44—C45—C46—C41	0.9 (4)
C3—C4—C5—C6	0.0	C42—C41—C46—C45	−1.2 (3)
C4—C5—C6—C7	0.0	C40—C41—C46—C45	176.6 (2)
C5—C6—C7—C2	0.0	C40—Sn2—C47—C48	136.09 (16)
C3—C2—C7—C6	0.0	C54—Sn2—C47—C48	−29.7 (2)
C1—C2—C7—C6	−178.8 (13)	O2—Sn2—C47—C48	57.29 (16)
C2—C1—C2'—C3'	153 (10)	Cl2—Sn2—C47—C48	−127.08 (16)
Sn1—C1—C2'—C3'	−116.1 (6)	Sn2—C47—C48—C49	56.6 (3)
C2—C1—C2'—C7'	−25 (9)	Sn2—C47—C48—C53	−124.4 (2)
Sn1—C1—C2'—C7'	66.4 (9)	C53—C48—C49—C50	1.9 (4)
C7'—C2'—C3'—C4'	0.0	C47—C48—C49—C50	−179.0 (2)
C1—C2'—C3'—C4'	−177.4 (13)	C48—C49—C50—C51	−1.9 (4)
C2'—C3'—C4'—C5'	0.0	C49—C50—C51—C52	0.1 (4)
C3'—C4'—C5'—C6'	0.0	C50—C51—C52—C53	1.5 (4)
C4'—C5'—C6'—C7'	0.0	C49—C48—C53—C52	−0.4 (4)
C5'—C6'—C7'—C2'	0.0	C47—C48—C53—C52	−179.4 (2)
C3'—C2'—C7'—C6'	0.0	C51—C52—C53—C48	−1.4 (4)
C1—C2'—C7'—C6'	177.5 (12)	C40—Sn2—C54—C55	13.7 (2)
C1—Sn1—C8—C9	169.36 (13)	C47—Sn2—C54—C55	178.34 (18)
C15—Sn1—C8—C9	−0.35 (17)	O2—Sn2—C54—C55	95.06 (19)
O1—Sn1—C8—C9	85.35 (14)	Cl2—Sn2—C54—C55	−84.48 (19)
Cl1—Sn1—C8—C9	−94.97 (14)	Sn2—C54—C55—C60	−107.8 (2)
Sn1—C8—C9—C14	−105.9 (2)	Sn2—C54—C55—C56	76.2 (3)
Sn1—C8—C9—C10	73.1 (2)	C60—C55—C56—C57	−0.5 (3)
C14—C9—C10—C11	−1.0 (3)	C54—C55—C56—C57	175.6 (2)
C8—C9—C10—C11	179.9 (2)	C55—C56—C57—C58	0.6 (4)
C9—C10—C11—C12	0.5 (4)	C56—C57—C58—C59	−0.3 (4)
C10—C11—C12—C13	0.3 (4)	C57—C58—C59—C60	−0.1 (4)
C11—C12—C13—C14	−0.5 (4)	C56—C55—C60—C59	0.1 (3)
C12—C13—C14—C9	0.0 (4)	C54—C55—C60—C59	−176.0 (2)
C10—C9—C14—C13	0.8 (3)	C58—C59—C60—C55	0.2 (4)
C8—C9—C14—C13	179.9 (2)	O2—P2—C61—C66	−114.19 (19)
C1—Sn1—C15—C16	−11.30 (18)	C73—P2—C61—C66	124.44 (19)
C8—Sn1—C15—C16	158.45 (14)	C67—P2—C61—C66	8.2 (2)
O1—Sn1—C15—C16	77.02 (15)	O2—P2—C61—C62	62.0 (2)
Cl1—Sn1—C15—C16	−107.59 (14)	C73—P2—C61—C62	−59.4 (2)
Sn1—C15—C16—C21	56.0 (2)	C67—P2—C61—C62	−175.61 (17)
Sn1—C15—C16—C17	−124.15 (18)	C66—C61—C62—C63	−1.0 (3)
C21—C16—C17—C18	−0.8 (3)	P2—C61—C62—C63	−177.34 (19)
C15—C16—C17—C18	179.3 (2)	C61—C62—C63—C64	0.1 (4)
C16—C17—C18—C19	0.2 (3)	C62—C63—C64—C65	0.9 (4)
C17—C18—C19—C20	0.5 (4)	C63—C64—C65—C66	−1.0 (4)
C18—C19—C20—C21	−0.6 (3)	C62—C61—C66—C65	0.9 (3)
C17—C16—C21—C20	0.7 (3)	P2—C61—C66—C65	177.00 (18)
C15—C16—C21—C20	−179.42 (19)	C64—C65—C66—C61	0.1 (4)
C19—C20—C21—C16	0.0 (3)	O2—P2—C67—C68	6.2 (2)
O1—P1—C22—C23	27.9 (2)	C73—P2—C67—C68	126.80 (18)
C28—P1—C22—C23	−94.7 (2)	C61—P2—C67—C68	−117.20 (18)
C34—P1—C22—C23	149.28 (19)	O2—P2—C67—C72	−171.92 (18)
O1—P1—C22—C27	−155.58 (19)	C73—P2—C67—C72	−51.3 (2)
C28—P1—C22—C27	81.8 (2)	C61—P2—C67—C72	64.7 (2)
C34—P1—C22—C27	−34.2 (2)	C72—C67—C68—C69	0.6 (3)
C27—C22—C23—C24	0.5 (4)	P2—C67—C68—C69	−177.55 (17)
P1—C22—C23—C24	177.2 (2)	C67—C68—C69—C70	−1.2 (3)
C22—C23—C24—C25	−0.2 (4)	C68—C69—C70—C71	0.7 (4)
C23—C24—C25—C26	−0.4 (4)	C69—C70—C71—C72	0.4 (4)
C24—C25—C26—C27	0.5 (4)	C70—C71—C72—C67	−1.0 (4)
C25—C26—C27—C22	−0.2 (4)	C68—C67—C72—C71	0.5 (3)
C23—C22—C27—C26	−0.4 (4)	P2—C67—C72—C71	178.57 (19)
P1—C22—C27—C26	−176.88 (19)	O2—P2—C73—C74	43.85 (19)
O1—P1—C28—C29	−124.5 (2)	C67—P2—C73—C74	−76.87 (19)
C34—P1—C28—C29	114.6 (2)	C61—P2—C73—C74	166.33 (17)
C22—P1—C28—C29	−3.3 (2)	O2—P2—C73—C78	−134.83 (19)
O1—P1—C28—C33	53.3 (2)	C67—P2—C73—C78	104.4 (2)
C34—P1—C28—C33	−67.6 (2)	C61—P2—C73—C78	−12.4 (2)
C22—P1—C28—C33	174.49 (18)	C78—C73—C74—C75	−0.1 (3)
C33—C28—C29—C30	0.6 (4)	P2—C73—C74—C75	−178.84 (18)
P1—C28—C29—C30	178.30 (18)	C73—C74—C75—C76	0.7 (3)
C28—C29—C30—C31	−0.7 (4)	C74—C75—C76—C77	−0.8 (4)
C29—C30—C31—C32	0.2 (4)	C75—C76—C77—C78	0.1 (4)
C30—C31—C32—C33	0.5 (4)	C76—C77—C78—C73	0.5 (3)
C31—C32—C33—C28	−0.7 (4)	C74—C73—C78—C77	−0.5 (3)
C29—C28—C33—C32	0.1 (3)	P2—C73—C78—C77	178.10 (18)
